# Lenvatinib plus pembrolizumab versus sunitinib for advanced renal cell carcinoma: Japanese patients from the CLEAR study

**DOI:** 10.1002/cam4.5483

**Published:** 2022-12-01

**Authors:** Masatoshi Eto, Toshio Takagi, Go Kimura, Satoshi Fukasawa, Satoshi Tamada, Yuji Miura, Mototsugu Oya, Naoto Sassa, Satoshi Anai, Masahiro Nozawa, Hideki Sakai, Rodolfo Perini, Wataru Yusa, Hiroki Ikezawa, Tomoyuki Narita, Yoshihiko Tomita

**Affiliations:** ^1^ Department of Urology Kyushu University Hospital Fukuoka Japan; ^2^ Department of Urology Tokyo Women's Medical University Hospital Tokyo Japan; ^3^ Department of Urology Nippon Medical School Hospital Tokyo Japan; ^4^ Prostate Center and Division of Urology Chiba Cancer Center Chiba Japan; ^5^ Department of Urology Bell‐land General Hospital Osaka Japan; ^6^ Department of Medical Oncology Toranomon Hospital Tokyo Japan; ^7^ Department of Urology Keio University Hospital Tokyo Japan; ^8^ Department of Urology Aichi Medical University Aichi Japan; ^9^ Department of Urology Nara Medical University Nara Japan; ^10^ Department of Urology Kindai University Hospital Osaka Japan; ^11^ Department of Urology Nagasaki University Hospital Nagasaki Japan; ^12^ Merck & Co., Inc. Rahway New Jersey USA; ^13^ Japan and Asia Clinical Development Department Oncology Business Group, Eisai Co., Ltd. Tokyo Japan; ^14^ Clinical Data Science Department, Medicine Development Center Eisai Co., Ltd. Tokyo Japan; ^15^ Lenvima Alliance Management Eisai Co., Ltd. Tokyo Japan; ^16^ Department of Urology, Department of Molecular Oncology Niigata University Graduate School of Medicine Niigata Japan

**Keywords:** CLEAR study, Japanese patients, lenvatinib, pembrolizumab, renal cell carcinoma

## Abstract

**Background:**

The phase 3 CLEAR study demonstrated statistically significantly improved efficacy with lenvatinib plus pembrolizumab versus sunitinib, including progression‐free survival and overall survival, in patients with previously untreated advanced renal cell carcinoma. This subset analysis investigated efficacy and safety in Japanese patients randomized to lenvatinib plus pembrolizumab or sunitinib in the CLEAR study.

**Methods:**

Progression‐free survival, overall survival, tumor response, and safety were assessed in Japanese patients with previously untreated advanced renal cell carcinoma randomized to receive lenvatinib plus pembrolizumab (*n* = 42) or sunitinib (*n* = 31). Efficacy outcomes were analyzed by independent imaging review per Response Evaluation Criteria in Solid Tumors, version 1.1.

**Results:**

Progression‐free survival was longer with lenvatinib plus pembrolizumab than with sunitinib (median, 22.1 vs. 10.9 months; hazard ratio, 0.39; 95% CI, 0.20–0.74). Median overall survival was not estimable in the lenvatinib plus pembrolizumab arm and 30.6 months in the sunitinib arm (HR, 1.20; 95% CI, 0.39–3.66). Overall survival adjusted for the imbalance of Memorial Sloan‐Kettering Cancer Center prognostic risk group favored lenvatinib plus pembrolizumab (hazard ratio, 0.67; 95% CI, 0.18–2.39). Objective response rate (69.0% vs. 45.2%; odds ratio, 2.71; 95% CI, 1.03–7.10) was higher and median duration of response (20.3 vs. 9.1 months) was longer with lenvatinib plus pembrolizumab versus sunitinib. Grade ≥ 3 treatment‐emergent adverse events occurred in 95.2% versus 87.1% of patients in the lenvatinib plus pembrolizumab versus sunitinib arms.

**Conclusions:**

These findings support lenvatinib plus pembrolizumab as a potential first‐line treatment for Japanese patients with advanced renal cell carcinoma.

## Graphical abstract

This subset analysis investigated efficacy and safety in Japanese patients with previously untreated advanced renal cell carcinoma randomized to receive lenvatinib plus pembrolizumab or sunitinib in the phase 3 CLEAR study. In general, efficacy favored lenvatinib plus pembrolizumab versus sunitinib. Findings were consistent with those of the overall CLEAR study population and support the combination as a potential first‐line treatment for Japanese patients with advanced renal cell carcinoma.

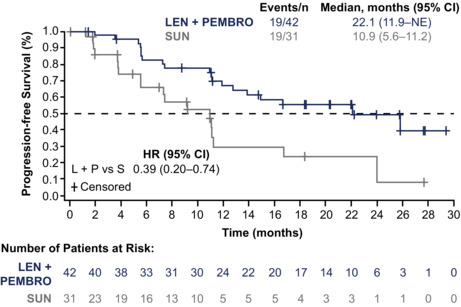

## 1 INTRODUCTION

Renal cell carcinoma (RCC), the urological cancer with the highest mortality rate, accounts for 2% of global cancer diagnoses and deaths.^1^ North America and Western Europe currently have the highest disease burden; however, RCC incidence in other geographic regions, including Asia, is projected to increase.^1^ Among Asian countries, Japan has one of the highest incidence rates for new cases of kidney cancer.^2^

Previous multicenter trials in patients with advanced RCC have shown more promising outcomes with tyrosine kinase inhibitors (TKIs) in combination with immune checkpoint inhibitors (ICI) than with TKI monotherapy.^3^, ^4^, ^5^, ^6^, ^7^, ^8^ Lenvatinib plus pembrolizumab, along with other TKI‐ICI combination regimens (e.g., axitinib plus pembrolizumab, cabozantinib plus nivolumab), are preferred by the National Comprehensive Cancer Network for the treatment of advanced RCC with clear cell histology.^9^ The open‐label, randomized, phase 3 CLEAR study demonstrated significantly improved progression‐free survival (PFS, primary endpoint; hazard ratio [HR], 0.39; 95% confidence interval [CI], 0.32–0.49, *p* < 0.001) and overall survival (OS; HR, 0.66; 95% CI, 0.49–0.88; *p* = 0.005) in patients with previously untreated advanced RCC who were randomized to receive lenvatinib plus pembrolizumab versus those randomized to receive sunitinib.^8^ Additionally, objective response rate (ORR) was higher in the lenvatinib plus pembrolizumab (71.0%) versus the sunitinib (36.1%) arm (odds ratio, 4.35; 95% CI, 3.16–5.97).^8^, ^10^ The safety profile of the combination was consistent with the known safety profile of lenvatinib and pembrolizumab monotherapies, and with previously reported safety profiles for the combination.^11^, ^12^, ^13^, ^14^

Previous studies of patients with advanced RCC treated with TKIs have revealed differences in drug tolerability profiles among Asian and non‐Asian populations. The incidence rates of certain adverse events (AEs), such as palmar‐plantar erythrodysesthesia syndrome and proteinuria, were reported to be higher in Asian populations compared with non‐Asian populations treated with sunitinib^15^, ^16^, ^17^, ^18^ or pazopanib.^15^, ^18^ These differences in safety profiles among Asian and non‐Asian populations of patients with metastatic RCC, along with the increasing incidence of kidney cancer in Japan,^2^ signal the need for studies that establish the efficacy and safety of treatments for advanced RCC in Japanese patients. Thus, we investigated efficacy and safety outcomes in the subset of Japanese patients from the CLEAR study who were randomized to receive lenvatinib plus pembrolizumab or sunitinib.

## 2 MATERIALS AND METHODS

### 2.1 Design and treatment

Full details of the multicenter, open‐label, randomized, three‐arm, phase 3 CLEAR trial have been previously published (ClinicalTrials.gov number, NCT02811861).^8^ Briefly, patients were randomized (1:1:1 ratio) to receive lenvatinib plus pembrolizumab, lenvatinib plus everolimus, or sunitinib. Randomization was stratified by geographic region (Western Europe and North America or the rest of the world) and Memorial Sloan‐Kettering Cancer Center (MSKCC) prognostic risk group (favorable, intermediate, or poor risk). In the lenvatinib plus pembrolizumab treatment arm, patients received lenvatinib at a dose of 20 mg orally once daily for each 21‐day treatment cycle and pembrolizumab at a dose of 200 mg intravenously on day 1 of each 21‐day cycle (with a maximum of 35 pembrolizumab treatments). In the sunitinib arm, patients received sunitinib at a dose of 50 mg orally once daily for 4 weeks followed by 2 weeks off treatment. The primary endpoint was PFS by independent imaging review (IIR) per Response Evaluation Criteria in Solid Tumors version 1.1 (RECIST v1.1). Key secondary endpoints were OS and ORR by IIR per RECIST v1.1, and other secondary endpoints included safety and health‐related quality of life. Key exploratory endpoints included duration of response (DOR). Tumor assessments were performed at screening and every 8 weeks from the date of randomization thereafter. AEs and serious AEs were monitored and recorded using the Common Terminology Criteria for Adverse Events, version 4.03.

In this subset analysis of the CLEAR study, we investigated efficacy (PFS, OS, ORR, and DOR) and safety (any grade, grade ≥ 3, and serious treatment‐emergent adverse events [TEAEs]) in Japanese patients randomized to receive lenvatinib plus pembrolizumab or sunitinib treatment arms. Tumors were assessed by RECIST v1.1 per IIR.

### 2.2 Patients

In the CLEAR study, patients were ≥18 years of age, had previously untreated RCC with a clear‐cell component, and had ≥1 measurable lesion according to RECIST v1.1. Other key inclusion criteria were a Karnofsky performance‐status score ≥70, adequately controlled blood pressure, and adequate organ function. Full details regarding patient inclusion and exclusion criteria have been published.^8^ All patients provided written informed consent, and the study protocol was approved by all relevant institutional review boards. The CLEAR study was conducted in accordance with the provisions of the Declaration of Helsinki and local laws.

### 2.3 Statistical analysis

This subset analysis in Japanese patients was conducted using data from the primary analysis of the CLEAR study (data cutoff date: August 28, 2020). Efficacy (PFS, OS, ORR, and DOR) was assessed in all Japanese patients who were randomized to receive lenvatinib plus pembrolizumab or sunitinib. PFS and OS were estimated using Kaplan–Meier estimates and two‐sided 95% CIs. Stratification was not performed in this subset analysis because of the limited sample size, and because the geographic region stratification factor could not be incorporated. Differences in PFS and OS between lenvatinib plus pembrolizumab and sunitinib treatment arms were evaluated with log‐rank tests, and Cox regression models with the Efron method for handling ties were used to estimate the HRs and 95% CIs. Two HRs were calculated for OS: an unadjusted HR (which included treatment group as a covariate) and an adjusted HR (which included treatment group and MSKCC risk group as covariates). The adjusted OS HR was calculated to account for the imbalance in MSKCC risk group between the lenvatinib plus pembrolizumab versus sunitinib treatment arms. Difference in ORR between lenvatinib plus pembrolizumab and sunitinib treatment arms was evaluated by odds ratio and 95% CIs. DOR (calculated for all patients with a confirmed response) was evaluated using the Kaplan–Meier method. Safety was assessed in all Japanese patients who received ≥1 dose of any study drug.

## 3 RESULTS

### 3.1 Patients

Of the 1069 randomized patients, 355 were assigned to the lenvatinib plus pembrolizumab arm and 357 were assigned to the sunitinib arm. Of those patients, there were 42 and 31 Japanese patients in the lenvatinib plus pembrolizumab and sunitinib arms, respectively. Baseline demographic and clinical characteristics in Japanese and overall patient populations are listed in Table 1. A larger percentage of Japanese patients had sarcomatoid features (14.3% vs. 3.2%) and no prior nephrectomy (38.1% vs. 16.1%) in the lenvatinib plus pembrolizumab arm compared with the sunitinib arm. Additionally, a smaller percentage of Japanese patients had programmed death ligand 1‐positive status in the lenvatinib plus pembrolizumab arm (28.6%) versus the sunitinib arm (38.7%). Other baseline characteristics were comparable among Japanese patients in lenvatinib plus pembrolizumab and sunitinib treatment arms.

**TABLE 1:** Baseline demographic and clinical characteristics

Patient characteristic	Lenvatinib + pembrolizumab		Sunitinib	
	Japanese (*n* = 42)	Overall^8^ (*n* = 355)	Japanese (*n* = 31)	Overall^8^ (*n* = 357)
Median age (range), years	67 (44–84)	64 (34–88)	63 (41–76)	61 (29–82)
Sex, male, %	78.6	71.8	77.4	77.0
Median weight (range), kg	60.3 (41.8–104.8)	79.2 (40.0–146.4)^a^	67.9 (40.5–89.7)	81.7 (40.5–173.0)^a^
KPS ≥90, %	97.6	83.1	93.5	82.4
Sarcomatoid features, %	14.3	7.9	3.2	5.9
MSKCC prognostic risk group, %				
Favorable/intermediate/poor	31.0/59.5/9.5	27.0/63.9/9.0	29.0/67.7/3.2	27.2/63.9/9.0
IMDC risk group, %				
Favorable/intermediate/poor	38.1/61.9/0.0	31.0/59.2/9.3	35.5/64.5/0.0	34.7/53.8/10.4
PD‐L1 status, %				
Positive (CPS ≥1)	28.6	30.1	38.7	33.3
Negative (CPS <1)	21.4	31.5	19.4	28.9
Not available	50.0	38.3	41.9	37.8
No prior nephrectomy, %	38.1	26.2	16.1	23.0

Abbreviations: CPS, combined positive score; IMDC, International Metastatic RCC Database Consortium; KPS, Karnofsky Performance Status; MSKCC, Memorial Sloan‐Kettering Cancer Center; PD‐L1, programmed death ligand 1.

^a^ Median weight and range of weight for the overall population was not reported in the primary manuscript.

### 3.2 Efficacy

**FIGURE 1:**
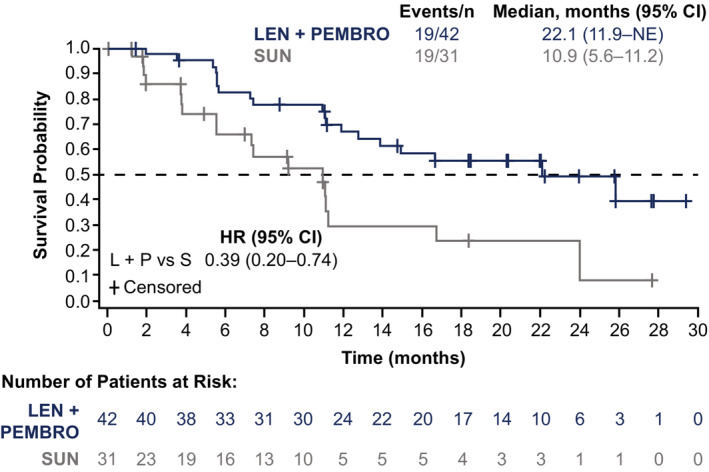
Progression‐free survival in Japanese patients by independent imaging review per RECIST v1.1. CI, confidence interval; HR, hazard ratio; L/LEN, lenvatinib; NE, not estimable; P/PEMBRO, pembrolizumab; RECIST v1.1, Response Evaluation Criteria In Solid Tumors version 1.1; S/SUN, sunitinib.

PFS as determined by IIR per RECIST v1.1 was longer in Japanese patients randomized to the lenvatinib plus pembrolizumab arm (median, 22.1 months; 95% CI, 11.9–not estimable [NE]) versus those randomized to the sunitinib arm (median, 10.9 months; 95% CI, 5.6–11.2), with an HR of 0.39 (95% CI, 0.20–0.74) (Figure 1). Median OS was not estimable (95% CI, NE–NE) in the lenvatinib plus pembrolizumab arm and 30.6 (95% CI, 24.8–30.6) months in the sunitinib arm; the unadjusted HR for OS was 1.20 (95% CI, 0.39–3.66) (Table 2; Figure S1). The OS HR adjusted by MSKCC risk group as a covariate was 0.67 (95% CI, 0.18–2.39). The ORR was 69.0% (with eight complete responses [CRs] and 21 partial responses [PRs]) in patients randomized to receive lenvatinib plus pembrolizumab and 45.2% (with two CRs and 12 PRs) in patients randomized to receive sunitinib (odds ratio, 2.71; 95% CI, 1.03–7.10) (Table 3). Median DOR in patients with an objective response was 20.3 months (95% CI, 13.1–NE) and 9.1 months (95% CI, 3.9–22.0) in lenvatinib plus pembrolizumab and sunitinib arms, respectively. One patient in the lenvatinib plus pembrolizumab arm and four patients in the sunitinib arm had progressive disease (Table 3).

**TABLE 2:** Overall survival in Japanese patients

Parameter	LEN + PEMBRO (*n* = 42)	SUN (*n* = 31)
Deaths, *n*	8	6
Median OS, months (95% CI)^a^	NE (NE–NE)	30.6 (24.8–30.6)
OS unadjusted HR (95% CI)^b^	1.20 (0.39–3.66)	
OS adjusted HR (95% CI)^c^	0.67 (0.18–2.39)	

Abbreviations: CI, confidence interval; HR, hazard ratio; LEN, lenvatinib; MSKCC, Memorial Sloan‐Kettering Cancer Center; NE, not estimable; OS, overall survival; PEMBRO, pembrolizumab; SUN, sunitinib.

^a^ 95% CIs estimated with a generalized Brookmeyer and Crowley method.

^b^ Hazard ratio based on a Cox proportional hazard model including treatment group as a factor, Efron method used for ties.

^c^ Hazard ratio based on a Cox proportional hazard model including treatment group and MSKCC risk group as factors, Efron method used for ties.

**TABLE 3:** Summary of tumor response by independent imaging review per RECIST v1.1 in Japanese patients

Parameter	Lenvatinib + pembrolizumab (*n* = 42)	Sunitinib (*n* = 31)
Best overall response, *n* (%)		
Complete response	8 (19.0)	2 (6.5)
Partial response	21 (50.0)	12 (38.7)
Stable disease	11 (26.2)	11 (35.5)
Progressive disease	1 (2.4)	4 (12.9)
Unknown/not evaluable	1 (2.4)	2 (6.5)
Objective response rate (CR + PR)		
*n* (%)	29 (69.0)	14 (45.2)
(95% CI)	(55.1–83.0)	(27.6–62.7)
Odds ratio (95% CI)	2.71 (1.03–7.10)	
Duration of objective response		
Patients with objective response, *n*	29	14
Median, months (95% CI)^a^	20.3 (13.1–NE)	9.1 (3.9–22.0)

Abbreviations: CI, confidence interval; CR, complete response; NE, not estimable; PR, partial response; RECIST v1.1, Response Evaluation Criteria In Solid Tumors version 1.1; SD, stable disease.

^a^ 95% CIs estimated with a generalized Brookmeyer and Crowley method.

**FIGURE 2:**
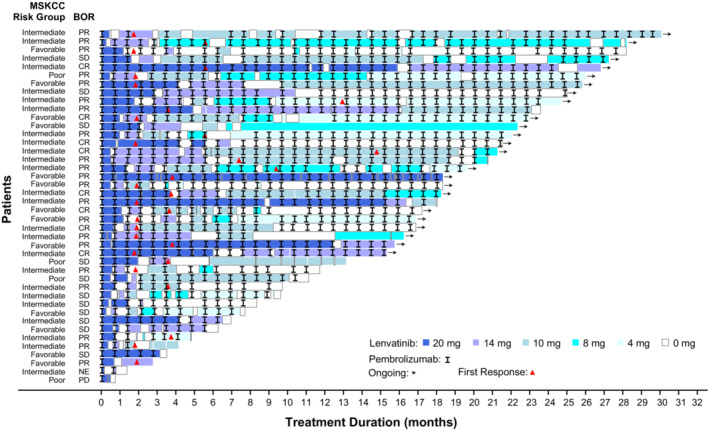
Duration of treatment and tumor response by independent imaging review per RECIST v1.1. BOR, best overall response; CR, complete response; MSKCC, Memorial Sloan‐Kettering Cancer Center; NE, not evaluable; PD, progressive disease; PR, partial response; RECIST v1.1, Response Evaluation Criteria In Solid Tumors version 1.1; SD, stable disease.

Patients' treatment durations and best overall tumor responses are shown in Figure 2. When comparing percent change in sums of diameters of target lesions from baseline to postbaseline nadir in 34 and 30 evaluable patients in the lenvatinib plus pembrolizumab and sunitinib arms, respectively, 12 (35.3%) patients in the lenvatinib plus pembrolizumab arm and four (13.3%) patients in the sunitinib arm had a maximum tumor reduction of ≥75% (Figure S2). Additionally, 28 (82.4%) patients in the lenvatinib plus pembrolizumab arm and 18 (60.0%) patients in the sunitinib arm had a maximum tumor reduction ≥30% (Figure S2).

### 3.3 Treatment exposure

The median dose intensity per patient was 9.47 mg/day and 25.45 mg/day in the lenvatinib plus pembrolizumab (for lenvatinib) and sunitinib arms, respectively (Table S1). In the lenvatinib plus pembrolizumab arm, the median number of administrations of pembrolizumab was 24.0 (range, 1–35). Median duration of treatment was 15.52 months for lenvatinib, 16.64 months for pembrolizumab, and 9.17 months for sunitinib. Overall median duration of treatment for lenvatinib plus pembrolizumab was 17.41 months.

### 3.4 Safety

An overview of TEAEs is shown in Table 4. Any‐grade treatment‐related TEAEs occurred in 100% of patients in both groups. Grade ≥ 3 TEAEs occurred in 95.2% of patients in the lenvatinib plus pembrolizumab arm and 87.1% of patients in the sunitinib arm (Table 5). Serious TEAEs occurred in 23 (54.8%) patients in the lenvatinib plus pembrolizumab arm (one patient had a fatal TEAE and 23 patients had nonfatal serious TEAEs) and 13 (41.9%) patients in the sunitinib arm (no patients had fatal TEAEs and 13 patients had nonfatal serious TEAEs) (Table 4). TEAEs leading to drug discontinuation occurred in 16 (38.1%) patients in the lenvatinib plus pembrolizumab treatment arm; 14 (33.3%) patients had a TEAE that led to lenvatinib discontinuation, 11 (26.2%) patients had a TEAE that led to pembrolizumab discontinuation, and seven (16.7%) patients had a TEAE that led to both lenvatinib and pembrolizumab discontinuation. In the sunitinib arm, six (19.4%) patients had a TEAE that led to drug discontinuation. In both lenvatinib plus pembrolizumab and sunitinib arms, each type of TEAE that lead to drug discontinuation was only experienced by one patient (Table S2). In the lenvatinib plus pembrolizumab arm, 39 (92.9%) patients had TEAEs leading to dose reduction of lenvatinib; in the sunitinib arm, 27 (87.1%) patients had TEAEs leading to dose reduction. TEAEs leading to dose interruption occurred in 36 (85.7%) patients in the lenvatinib plus pembrolizumab arm; within that arm, 32 (76.2%) patients had TEAEs leading to lenvatinib interruption, 28 (66.7%) patients had TEAEs leading to pembrolizumab interruption, and 18 (42.9%) patients had TEAEs leading to both lenvatinib and pembrolizumab interruption. In the sunitinib arm, 24 (77.4%) patients had TEAEs leading to dose interruption (Table 4).

**TABLE 4:** Overview of TEAEs and drug modifications

Category, *n* (%)	Lenvatinib + pembrolizumab (*n* = 42)	Sunitinib (*n* = 31)
Patients with any treatment‐related TEAEs	42 (100.0)	31 (100.0)
Patients with grade 3/4/5 TEAEs	37 (88.1)/2 (4.8)/1 (2.4)	20 (64.5)/7 (22.6)/0
Patients with any serious TEAEs^a^	23 (54.8)	13 (41.9)
Patients with any fatal TEAEs	1 (2.4)	0
Patients with any nonfatal serious TEAEs	23 (54.8)	13 (41.9)
Patients with TEAEs leading to discontinuation	16 (38.1)	6 (19.4)
LEN	14 (33.3)	—
PEMBRO	11 (26.2)	—
LEN + PEMBRO	7 (16.7)	—
Patients with TEAEs leading to dose reduction^b^	39 (92.9)	27 (87.1)
LEN	39 (92.9)	—
Patients with TEAEs leading to dose interruption	36 (85.7)	24 (77.4)
LEN	32 (76.2)	—
PEMBRO	28 (66.7)	—
LEN + PEMBRO	18 (42.9)	—

Abbreviation: TEAE, treatment‐emergent adverse event.

^a^ One patient developed both a nonfatal (pneumocystis pneumonia) and a fatal (cardiopulmonary arrest) TEAE.

^b^ Dose reductions of pembrolizumab were not permitted according to the study protocol.

**TABLE 5:** TEAEs ≥30% in any treatment group by preferred term

Preferred term, *n* (%)	Lenvatinib + pembrolizumab (*n* = 42)		Sunitinib (*n* = 31)	
	Any grade	Grade ≥3	Any grade	Grade ≥3
Patients with any TEAEs^a^	42 (100.0)	40 (95.2)	31 (100.0)	27 (87.1)
Diarrhea	24 (57.1)	6 (14.3)	14 (45.2)	1 (3.2)
Hypertension	24 (57.1)	8 (19.0)	15 (48.4)	8 (25.8)
Hypothyroidism	26 (61.9)	1 (2.4)	11 (35.5)	0
Decreased appetite	18 (42.9)	3 (7.1)	9 (29.0)	0
Stomatitis	18 (42.9)	1 (2.4)	11 (35.5)	1 (3.2)
Dysphonia	22 (52.4)	0	5 (16.1)	0
Weight decreased	15 (35.7)	6 (14.3)	0	0
Proteinuria	23 (54.8)	6 (14.3)	8 (25.8)	3 (9.7)
Palmar‐plantar erythrodysesthesia syndrome	28 (66.7)	5 (11.9)	20 (64.5)	1 (3.2)
Constipation	13 (31.0)	0	5 (16.1)	0
Pyrexia	11 (26.2)	0	13 (41.9)	1 (3.2)
Dysgeusia	10 (23.8)	0	17 (54.8)	0
Platelet count decreased	10 (23.8)	0	21 (67.7)	10 (32.3)
Malaise	14 (33.3)	0	12 (38.7)	0
White blood cell count decreased	1 (2.4)	0	12 (38.7)	4 (12.9)

Abbreviation: TEAE, treatment‐emergent adverse event.

^a^ Alanine aminotransferase/aspartate aminotransferase increased in 4.8%/7.1% (grade 3: 0%/0%) of patients in the lenvatinib plus pembrolizumab arm, and 16.1%/12.9% (grade 3: 9.7%/3.2%) in the sunitinib arm.

A summary of frequently occurring TEAEs (ie, those occurring in ≥30% of patients in any treatment group) in Japanese patients who received lenvatinib plus pembrolizumab or sunitinib is shown in Table 5. The most frequent any‐grade TEAEs in the lenvatinib plus pembrolizumab arm were palmar‐plantar erythrodysesthesia syndrome (28 [66.7%] patients), hypothyroidism (26 [61.9%] patients), diarrhea (24 [57.1%] patients), and hypertension (24 [57.1%] patients). The most frequent any‐grade TEAEs in the sunitinib arm were platelet count decreased (21 [67.7%] patients), palmar‐plantar erythrodysesthesia syndrome (20 [64.5%] patients), dysgeusia (17 [54.8%] patients), and hypertension (15 [48.4%]) patients. The most frequent grade ≥3 TEAEs in the lenvatinib plus pembrolizumab arm were hypertension and lipase increased (eight [19.0%] patients each). The most frequent grade ≥ 3 TEAEs in the sunitinib arm were platelet count decreased (10 [32.3%] patients) and hypertension (eight [25.8%] patients).

Immune‐mediated AEs occurred in 31 (73.8%) patients in the lenvatinib plus pembrolizumab arm. Seven (16.7%) patients were treated with high‐dose corticosteroids to manage immune‐mediated AEs. Of these patients, two (4.8%) received high‐dose corticosteroids for 14 or more consecutive days, and none of the patients received high‐dose corticosteroids for 30 or more consecutive days. The most common immune‐mediated AEs treated with high‐dose corticosteroids were adrenal insufficiency and hypothyroidism (4.8% each).

## 4 DISCUSSION

Our subset analysis of Japanese patients randomized to receive lenvatinib plus pembrolizumab or sunitinib in the CLEAR study demonstrated clinical benefit consistent with the observations in the overall CLEAR study population.^8^ PFS benefit for lenvatinib plus pembrolizumab versus sunitinib was comparable between Japanese patients (HR for disease progression or death, 0.39) and the overall patient population (HR for disease progression or death, 0.39; *p* < 0.001). Additionally, when adjusted for MSKCC risk group, the OS benefit in Japanese patients (HR for death, 0.67) was comparable to the benefit in the overall population of patients receiving lenvatinib plus pembrolizumab versus sunitinib (HR, 0.66; *p* = 0.005). A similar ORR benefit for lenvatinib plus pembrolizumab versus sunitinib was also observed among the Japanese subset of patients (odds ratio 2.71) and the overall population (odds ratio 4.35).^8^, ^10^ Importantly, in the Japanese subset, eight (19.0%) and 21 (50.0%) patients randomized to the lenvatinib plus pembrolizumab arm had a CR and a PR, respectively (vs. 16.1% of patients with CRs and 54.9% of patients with PRs in the overall CLEAR study population).

Notably, median dose intensity was lower in Japanese patients (lenvatinib, 9.47 mg/day; sunitinib, 25.45 mg/day) than in the overall population (lenvatinib, 13.93 mg/day; sunitinib, 41.59 mg/day). These differences, which may be due to the careful management of specific TKI‐related toxicities that occurred more frequently in Japanese patients than in the overall population (discussed in detail below), should be considered when comparing efficacy and safety among the Japanese and overall CLEAR study populations. Importantly, although lower median dose intensity was observed for Japanese patients from the CLEAR trial, a previous phase 1 study of lenvatinib in Japanese patients with advanced solid tumors showed that lenvatinib was tolerated up to 24 mg once daily with no dose‐limiting toxicities reported at this dose.^19^ Additionally, studies with lenvatinib have reported similar pharmacokinetic profiles in Japanese and non‐Japanese patients with solid tumors,^19^, ^20^ thereby supporting the treatment of Japanese patients at the recommended starting dose of lenvatinib, with dose modifications used as needed to manage toxicities.

The overall safety profile of lenvatinib plus pembrolizumab in this study was manageable and generally consistent with the known safety profile of the combination.^8^, ^11^, ^12^, ^14^ The most frequent TEAEs in the lenvatinib plus pembrolizumab arm were similar among the Japanese and overall CLEAR study populations.^8^ In Japanese patients, the most frequent any‐grade TEAEs were palmar‐plantar erythrodysesthesia syndrome (66.7%), hypothyroidism (61.9%), diarrhea (57.1%), and hypertension (57.1%). Comparably, in the overall population, the most frequent any‐grade TEAEs were diarrhea (61.4%), hypertension (55.4%), hypothyroidism (47.2%), and decreased appetite (40.3%). The percentage of patients with any immune‐mediated AEs was higher in Japanese patients randomized to receive lenvatinib plus pembrolizumab (73.8%) than for that in the overall population (60.8%); however, a similar number of patients in the Japanese subset (16.7%) versus the overall population (14.8%) were treated with high‐dose corticosteroids for any duration to manage immune‐mediated AEs.^8^ Additionally, a similar number of patients in both populations received corticosteroids for ≥14 consecutive days to manage immune‐mediated AEs (Japanese patients: 4.8%; overall CLEAR study population: 5.1%). Notably, one of the most common immune‐mediated AEs treated with high‐dose corticosteroids in both the Japanese and overall populations was hypothyroidism (4.8% and 2.8%, respectively).

Although safety profiles were generally similar among Japanese and overall populations of patients who received lenvatinib plus pembrolizumab, some important differences in the frequency of specific TEAEs among Japanese patients and the overall CLEAR study population emerged.^8^ Notably, among patients who received lenvatinib plus pembrolizumab, the incidences of palmar‐plantar erythrodysesthesia syndrome (66.7% vs. 28.7%), proteinuria (54.8% vs. 29.5%), dysphonia (52.4% vs. 29.8%), and hypothyroidism (61.9% vs. 47.2%) were higher in the Japanese subset from CLEAR versus the overall population, respectively. A higher incidence of palmar‐plantar erythrodysesthesia syndrome was also observed in Japanese patients randomized to receive sunitinib (64.5% in Japanese patients vs. 37.4% in the overall population). Similarly, previous trials investigating TKIs (sunitinib or pazopanib) in patients with metastatic RCC have reported higher rates of any grade^15^, ^18^ and grade 3–4^18^ proteinuria and any‐grade palmar‐plantar erythrodysesthesia syndrome^15^, ^16^, ^17^ in Asian versus non‐Asian patient populations.

A higher incidence of treatment‐emergent proteinuria of any grade was also observed in the phase 3 Study 309/KEYNOTE‐775 in Japanese patients with advanced endometrial cancer who received lenvatinib plus pembrolizumab (63.5%)^21^, ^22^ as compared with those in the overall population who received lenvatinib plus pembrolizumab (28.8%).^23^ Additionally, a subset analysis of the phase 3 SELECT study of lenvatinib versus placebo in patients with radioiodine‐refractory differentiated thyroid cancer^20^ demonstrated higher incidences of these two TEAEs in the Japanese subset of patients who received lenvatinib versus those in the overall population who received lenvatinib (any‐grade proteinuria: 63.3% vs. 31.0%; any‐grade palmar‐plantar erythrodysesthesia syndrome: 70.0% vs. 31.8%). Our results, together with these reported differences in Japanese patients with advanced RCC and other cancer types, signal the need for careful management of TKI‐related toxicities such as proteinuria and palmar‐plantar erythrodysesthesia syndrome in Japanese patients with advanced RCC.

Recently, a randomized phase 2 trial (Study 218) comparing two starting doses of lenvatinib (14 mg vs. 18 mg; both in combination with everolimus) in patients with advanced RCC, showed that the lower starting dose of lenvatinib (in combination with everolimus) was not noninferior to the higher starting dose, based on a test of ORR at week 24 in the 14 mg versus the 18 mg arm.^24^ In the current study, the majority of patients had a dose reduction. First responses (CR or PR) were observed early (within 4 months of treatment initiation) in the majority of Japanese patients (22 of 29 patients) randomized to receive lenvatinib plus pembrolizumab who had a CR or a PR, and the majority of patients were receiving 20 mg or 14 mg of lenvatinib at the time of first response. These observations, together with the results of Study 218, support that initiating lenvatinib at the recommended 20 mg starting dose and reducing the dose as needed can facilitate tumor reduction, and may also signal the importance of close monitoring of patients to manage adverse events so that the patients can remain on treatment and still maintain therapeutic benefit.

### 4.1 Limitations

Caution should be used when interpreting the results due to the small sample size and lack of stratification of Japanese patients included in this subset analysis. To help address the lack of stratification, the adjusted OS analysis accounted for the imbalance of MSKCC risk group between treatment arms.

## 5 CONCLUSION

The rising incidence of kidney cancer in Japan^2^ signals the increasing need for effective treatment options. Safety and efficacy data of lenvatinib plus pembrolizumab were generally consistent with those of the overall population; however, some important differences in the frequency of specific TEAEs emerged among Japanese patients and the overall CLEAR study population. Our subset analysis showed that, with close monitoring of patients to manage adverse events (e.g., lenvatinib dose reductions as needed), lenvatinib at the recommended 20 mg starting dose plus pembrolizumab (200 mg intravenously once every 3 weeks) demonstrated therapeutic benefit in Japanese patients with advanced RCC in the CLEAR study. Together, the results of this subset analysis are supportive of lenvatinib plus pembrolizumab as a potential first‐line treatment for Japanese patients with advanced RCC.

## AUTHOR CONTRIBUTIONS

**Masatoshi Eto:** Conceptualization (equal); investigation (equal); writing – original draft (lead); writing – review and editing (lead). **Toshio Takagi:** Investigation (equal); writing – original draft (equal); writing – review and editing (equal). **Go Kimura:** Investigation (equal); writing – original draft (equal); writing – review and editing (equal). **Satoshi Fukasawa:** Investigation (equal); writing – original draft (equal); writing – review and editing (equal). **Satoshi Tamada:** Investigation (equal); writing – original draft (equal); writing – review and editing (equal). **Yuji Miura:** Investigation (equal); writing – original draft (equal); writing – review and editing (equal). **Mototsugu Oya:** Investigation (equal); writing – original draft (equal); writing – review and editing (equal). **Naoto Sassa:** Investigation (equal); writing – original draft (equal); writing – review and editing (equal). **Satoshi Anai:** Investigation (equal); writing – original draft (equal); writing – review and editing (equal). **Masahiro Nozawa:** Investigation (equal); writing – original draft (equal); writing – review and editing (equal). **Hideki Sakai:** Investigation (equal); writing – original draft (equal); writing – review and editing (equal). **Rodolfo Perini:** Conceptualization (equal); writing – original draft (equal); writing – review and editing (equal). **Wataru Yusa:** Conceptualization (equal); writing – original draft (equal); writing – review and editing (equal). **Hiroki Ikezawa:** Data curation (equal); formal analysis (equal); writing – original draft (equal); writing – review and editing (equal). **Tomoyuki Narita:** Conceptualization (equal); writing – original draft (equal); writing – review and editing (equal). **Yoshihiko Tomita:** Investigation (equal); writing – original draft (equal); writing – review and editing (equal).

## FUNDING INFORMATION

This study was sponsored by Eisai Inc., Nutley, NJ, USA, and Merck Sharp & Dohme LLC, a subsidiary of Merck & Co., Inc., Rahway, NJ, USA. Medical writing support was funded by Eisai Inc., Nutley, NJ, USA, and Merck Sharp & Dohme LLC, a subsidiary of Merck & Co., Inc., Rahway, NJ, USA.

## CONFLICT OF INTEREST

ME: Lecture fees, honoraria, or other fees: ONO, Takeda, Novartis, Pfizer, Bristol Myers Squibb, Janssen, MSD, Merck, AstraZeneca, Eisai; research funds: Bristol Myers Squibb, ONO, Eisai, Taiho, MSD; scholarship endowments or research grants: Sanofi, Bayer, Astellas, ONO, Takeda; editorial board member: *Cancer Science*. TT: Lecture fees, honoraria, or other fees: Takeda, Ono, Merck. GK: Lecture fees, honoraria, or other fees: Ono, Bristol Myers Squibb, Bayer. SF: No disclosures to report. ST: Lecture fees, honoraria, or other fees: Merck Biophama, Takeda, MSD, Pfizer. YM: Research funds: Ono, MSD. MO: Lecture fees, honoraria, or other fees: Pfizer, Novartis, Bayer, Ono, Bristol Myers Squibb, Takeda, MSD, Merck, Eisai; manuscript fee: Pfizer; scholarship endowments or research grants: Ono, Takeda; editorial board member: *Cancer Science*. NS: Lecture fees, honoraria, or other fees: Ono, Bristol Myers Squibb, MSD, Eisai. SA: No disclosures to report. MN: No disclosures to report. HS: No disclosures to report. RP: Employee of Merck Sharp & Dohme LLC, a subsidiary of Merck & Co., Inc., Rahway, NJ, USA. WY: Employee of Eisai Co., Ltd. HI: Employee of Eisai Co., Ltd. TN: Employee of Eisai Co., Ltd. YT: Lecture fees: Ono, BMS, Merck, Takeda; Research funds: Ono, Chugai.

## ETHICS STATEMENT

*Approval of the research protocol by an Institutional Reviewer Board*: The study protocol was approved by all relevant institutional review bodies. The CLEAR study was conducted in accordance with the provisions of the Declaration of Helsinki and local laws. *Informed consent*: All patients provided written informed consent. *Registry and the Registration No. of the trial*: ClinicalTrials.gov Identifier: NCT02811861. *Animal Studies*: N/A.

## Supporting information


Appendix S1

## Data Availability

The data will not be available for sharing at this time because the data are commercially confidential. However, Eisai will consider written requests to share the data on a case‐by‐case basis.
