# Supplementary material for: Lenvatinib plus pembrolizumab versus sunitinib for advanced renal cell carcinoma: Japanese patients from the CLEAR study
Source: Cancer Med. 2022 Dec 1;12(6):6902–12. doi: 10.1002/cam4.5483 (PMC10067092; doi:10.1002/cam4.5483)

**SUPPORTING INFORMATION**

**SUPPLEMENTAL TABLE 1** Treatment exposure in Japanese patients^a^

| **Parameter** | **Lenvatinib + pembrolizumab** (n = 42) | **Sunitinib** (n = 31) |
| --- | --- | --- |
| Total dose per patient ^b^ (mg)  Mean (SD)  Median  Range | 4274.4 (3250.18)  3422.0  136, 13348 | 6379.0 (4794.75)  4975.0  500, 15925 |
| Dose intensity per patient ^b^ (mg/day)  Mean (SD)  Median  Range | 10.24 (4.737)  9.47  2.5, 20.0 | 29.33 (9.484)  25.45  17.1, 50.0 |
| Dose received as percentage of planned starting dose per patient, ^b^ %  Mean (SD)  Median  Range | 51.19 (23.683)  47.36  12.6, 100.0 | 58.65 (18.969)  50.89  34.3, 100.0 |

^a^In the overall populations of the lenvatinib plus pembrolizumab and sunitinib arms, the respective median doses per patient were 6440.0 mg (for lenvatinib) and 6637.5 mg (for sunitinib), and the median dose intensities per patient were 13.93 mg/day (for lenvatinib) and 41.59 mg/day (for sunitinib).
^b^For lenvatinib or sunitinib only (dose reductions were not permitted for pembrolizumab).
Abbreviation: SD, standard deviation.

**SUPPLEMENTAL TABLE 2** TEAEs leading to treatment discontinuation of any study drug in Japanese patients (TEAEs experienced by ≥1 patient in either treatment arm are shown)^a^

| **Preferred Term**, n (%) | **Lenvatinib + pembrolizumab**  (n = 42) | | **Sunitinib** (n = 31) | |
| --- | --- | --- | --- | --- |
|  | Overall | Grade ≥3 | Overall | Grade ≥3 |
| Patients with any TEAEs leading to treatment discontinuation | 16 (38.1) | 9 (21.4) | 6 (19.4) | 6 (19.4) |
| Myocardial infarction | 1 (2.4) | 1 (2.4) | 0 | 0 |
| Cardiac failure acute | 1 (2.4) | 1 (2.4) | 0 | 0 |
| Cardio-respiratory arrest | 1 (2.4) | 1 (2.4) | 0 | 0 |
| Diarrhea | 1 (2.4) | 0 | 0 | 0 |
| Immune-mediated pancreatitis | 1 (2.4) | 0 | 0 | 0 |
| Small intestinal hemorrhage | 0 | 0 | 1 (3.2) | 1 (3.2) |
| Pyrexia | 0 | 0 | 1 (3.2) | 1 (3.2) |
| Immune-mediated hepatitis | 1 (2.4) | 1 (2.4) | 0 | 0 |
| Hepatic function abnormal | 1 (2.4) | 0 | 0 | 0 |
| Pneumocystis jirovecii pneumonia | 1 (2.4) | 1 (2.4) | 0 | 0 |
| Sepsis | 0 | 0 | 1 (3.2) | 1 (3.2) |
| Lipase increased | 1 (2.4) | 1 (2.4) | 0 | 0 |
| Electrocardiogram QT prolonged | 1 (2.4) | 0 | 0 | 0 |
| Platelet count decreased | 0 | 0 | 1 (3.2) | 1 (3.2) |
| Cystitis | 1 (2.4) | 0 | 0 | 0 |
| Cancer pain | 1 (2.4) | 0 | 0 | 0 |
| Chronic myeloid leukaemia | 0 | 0 | 1 (3.2) | 1 (3.2) |
| Paralysis recurrent laryngeal nerve | 0 | 0 | 1 (3.2) | 1 (3.2) |
| Renal failure | 1 (2.4) | 1 (2.4) | 0 | 0 |
| Pneumonitis | 1 (2.4) | 0 | 0 | 0 |
| Lung disorder | 1 (2.4) | 1 (2.4) | 0 | 0 |
| Rash | 1 (2.4) | 1 (2.4) | 0 | 0 |
| Drug eruption | 1 (2.4) | 0 | 0 | 0 |
| Erythema multiforme | 1 (2.4) | 1 (2.4) | 0 | 0 |
| Palmar-plantar erythrodysaesthesia syndrome | 1 (2.4) | 1 (2.4) | 0 | 0 |

^a^Percentages are based on the total number of patients in the Safety Analysis Set (patients who received ≥1 dose of any study drug) within the relevant treatment group. MedDRA preferred terms “Neoplasm Progression,” “Malignant Neoplasm Progression,” and “Disease Progression” not related to the study drug are excluded. Patients with 2 or more TEAEs reported in the same preferred term are counted only once within that preferred term. Adverse Events terms were coded using MedDRA version 23.0. Adverse events were graded using CTCAE version 4.03.
Abbreviations: CTCAE, Common Terminology Criteria for Adverse Events; MEDRA, Medical Dictionary for Regulatory Activities; TEAE, treatment-emergent adverse event.

**SUPPLEMENTAL FIGURE 1** Overall survival in Japanese patients.^a a^Medians are estimated by the Kaplan-Meier method, and the 95% CIs are estimated with a generalized Brookmeyer and Crowley method. Hazard ratio is based on a Cox Proportional Hazard Model including treatment group as a factor, Efron method is used for ties. CIs, confidence intervals; HR, hazard ratio; L+P, lenvatinib + pembrolizumab; LEN, lenvatinib; PEMBRO, pembrolizumab; S, sunitinib; SUN, sunitinib; NE, not estimable


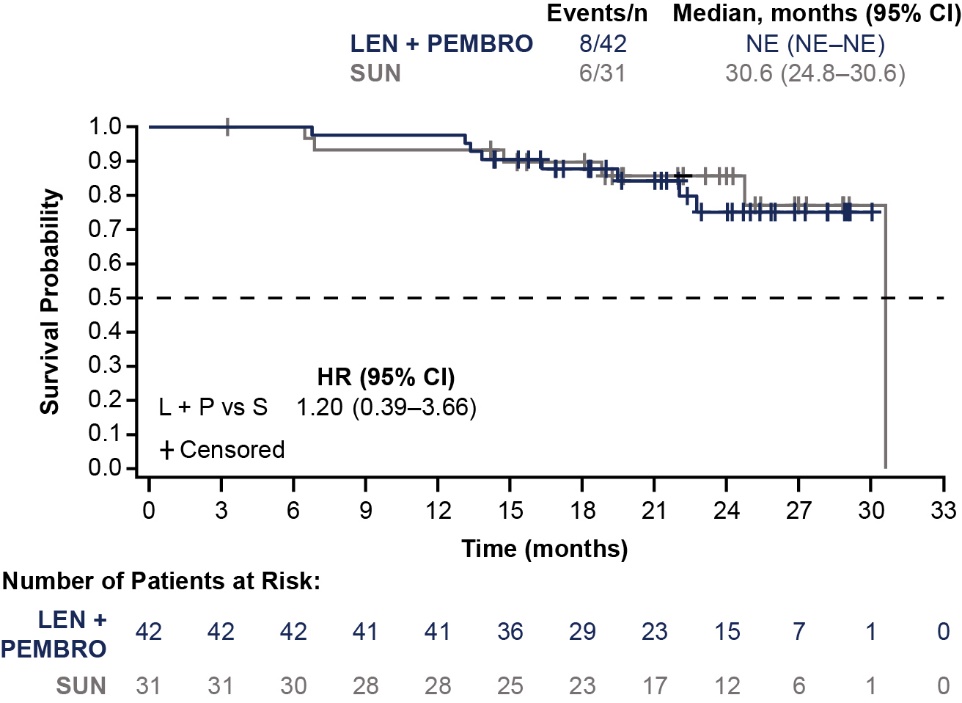


**SUPPLEMENTAL FIGURE 2** Percent changes in sums of diameters of target lesions from baseline to postbaseline nadir by independent review committee per RECIST v1.1.^a a^Includes patients with both baseline and at least one postbaseline target lesion assessment. LEN, lenvatinib; PEMBRO, pembrolizumab; RECIST v1.1, Response Evaluation Criteria In Solid Tumors version 1.1; SUN, sunitinib


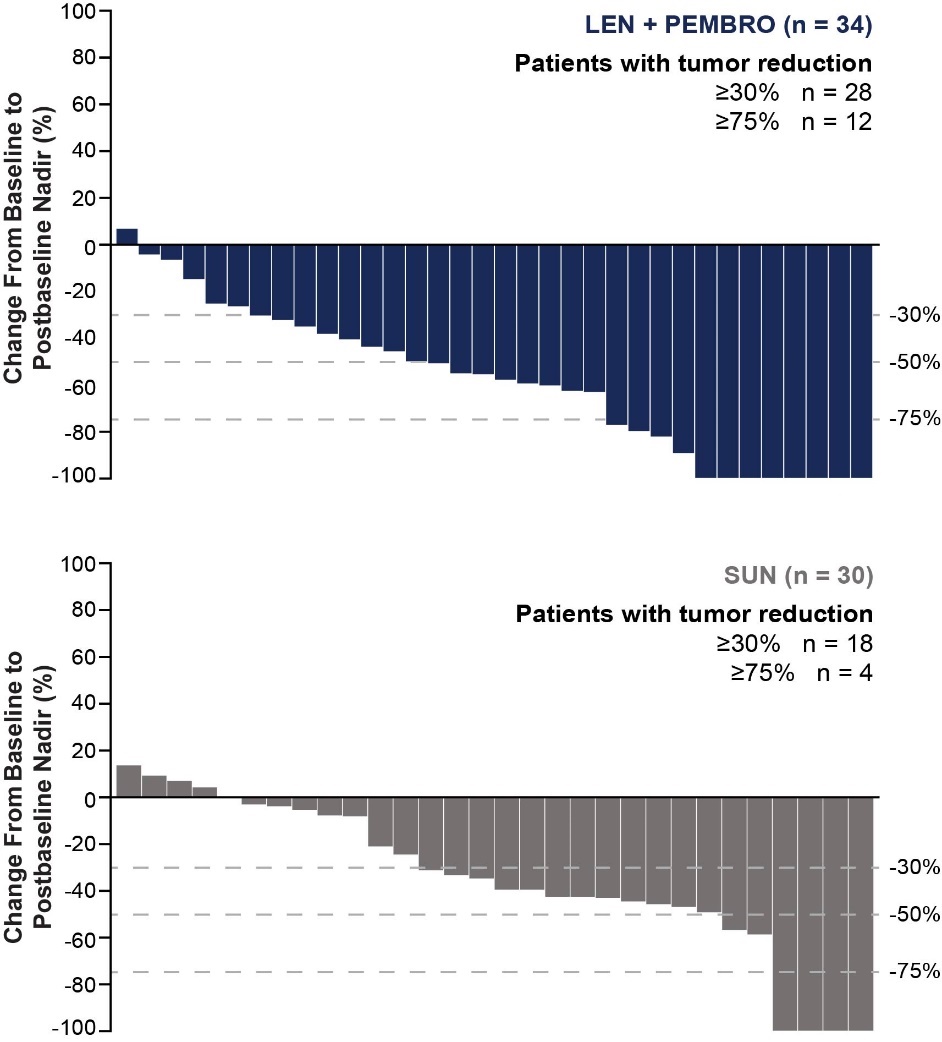

Supplement: Supplementary file 1 — Appendix S1 [file CAM4-12-6902-s001.docx]
